# A panel discussion on AI for science: the opportunities, challenges and reflections

**DOI:** 10.1093/nsr/nwae119

**Published:** 2024-03-26

**Authors:** Weijie Zhao

## Abstract

Artificial intelligence (AI) tools are changing the way we do science. AlphaFold basically solved the conundrum of protein structure prediction; DeepMD greatly improved the efficiency and accuracy of molecular simulations; and the emerging large language models such as ChatGPT are opening up more possibilities for scientific applications. In this panel, five experts from China and the US discussed the concept, development, bottlenecks and opportunities of AI for Science (AI4S), as well as their understanding of the relationship between AI and science.

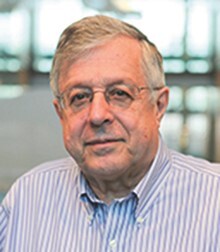

Roberto Car

Professor at Department of Chemistry, Princeton University, USA

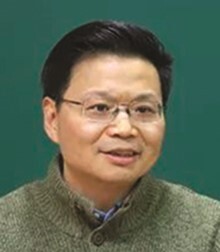

Weinan E

Professor at School of Mathematical Sciences, Peking University, China; AI for Science Institute, Beijing, China

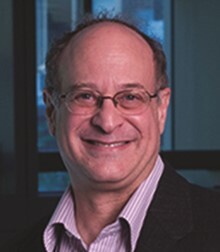

David Srolovitz

Professor at Department of Mechanical Engineering, University of Hong Kong, China

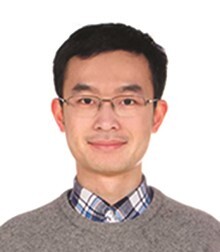

Han Wang

Professor at the Institute of Applied Physics and Computational Mathematics, Chinese Academy of Sciences, China

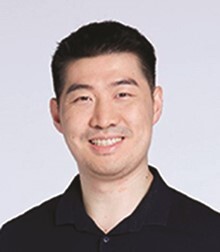

Linfeng Zhang (Chair)

Chief scientific officer of DP Technology, China; AI for Science Institute, Beijing, China

## THE EMERGING AI4S TOOLS

**Zhang:** Let's start with a basic question: what is ‘AI for Science’?

**E:** AI for Science is a new research paradigm in which we use AI tools to enhance our ability to do scientific research, similar in spirit to the way we use computers to help us. Specifically, AI-based algorithms can greatly improve the efficiency and accuracy for first-principle-based modeling. AI can also help to improve the way we do experiments, by providing new experimental designs, more accurate and efficient experimental characterization algorithms, and even new experimental apparatus. In addition, the work-flow and the open-source collaborative spirit in AI research is also an inspiration for scientific research.

**Car:** In my view, AI provides a set of tools, machine learning and deep neural network as representations, that can facilitate scientific discovery. To do that, a number of specific tools need to be developed. I would like to quote three examples in my research domain.

First, AI can bridge the gap between quantum mechanics and classical coarse-grained models. In this endeavor, AI greatly enhanced the accuracy and the time/space range of the molecular coarse-grained models that are used for molecular simulation. This enhancement seems to be just a quantitative effect, but in these domains, quantity is also quality. Just as Philip Anderson noted, more is different. And the approach is already leading to new discoveries.

The second example is the design of new materials and molecules with desired properties. I am not directly working in that domain, but I know that there are many people doing it and AI is making it possible to harness a large amount of data—from experiment, theory and simulation—to suggest which materials or molecules may be better suited for certain purposes.

The third example is that AI may be used to analyse experimental data to improve the selectivity of the probes by enhancing the signal-to-noise ratio with software.

**Wang:** Besides the examples mentioned by Roberto, AI tools are also changing how we process scientific data. Especially, large language models can extract knowledge and key points from scientific figures and literature very efficiently.

Moreover, AI is changing the way of developing scientific software by helping us to automatically generate code, to better detect bugs, and to give us suggestions to improve the efficiency of the code. All these AI tools have greatly improved the efficiency of scientific research.

**Srolovitz:** I agree that AI will greatly change our traditional way of reading the literature. It used to be that when we wanted to make an exhaustive study of the literature of an area, we get a graduate student to do that and draft a review paper. But in the future, we may ask the large language models to do that. The literature is so big that I think human beings are not capable of digesting that quantity of information.

AI for Science is a new research paradigm in which we use AI tools to enhance our ability to do scientific research.

—Weinan E

## NEW OPPORTUNITIES FROM LARGE LANGUAGE MODELS

**Zhang:** Except for reading papers, what new possibilities will the large language models bring to AI4S?

**Srolovitz:** We were actually playing with these models. We tried something that has been reported by others: to ask the model to predict whether a material is going to be glass, amorphous or crystalline. We gave it 15 examples and asked it to classify another 10, and it achieved an accuracy of 70% in seconds. That is very interesting. And what is more interesting is that, when you ask the model ‘why did you give these results?’, it gives you reasons. They weren't necessarily very good reasons, but they worked.

For the development of these models, prompt engineering will be an interesting direction that can help us to better guide the models and make them able to do more amazing things.

**Wang:** Generative model techniques would be very helpful for scientific research. For instance, a lot of scientific problems need sampling of high-dimensional probability distributions, which is basically a generation problem. There are already successful examples of using the generative tools such as diffusion models and generative adversarial networks (GANs) to generate samples in high-dimensional distributions.

Another example is that the conditional diffusion model can be used to design molecules to have certain properties in given conditions. This opens a new possibility for solving the problem of molecule and materials design.

**Zhang:** A good starting point for these models to benefit scientific research is their ability to read images. For example, they may be used to read electron microscopy photographs and generate structures from the images.

Currently we are trying to develop new tools to improve the ability of large language models to understand scientific literature. Especially, as molecules are expressed in different forms such as text, formula or image, the current models are not very good at combining this information for a thorough understanding.

**E:** It could potentially give suggestions for new questions, and new ideas. It also helps to bring different scientific disciplines together.

## AI VS. SCIENCE: ANSWER VS. UNDERSTANDING

**Srolovitz:** I would like to talk about the relationship between AI and science a little bit more philosophically. I had thought of the AI approach as anti-scientific, because it often was about getting answers, but not about getting understanding. But the mission of science is to develop understanding.

I've changed my perspective, though. The reason is that I started to see that when we have a lot of trustable answers, these answers can provide valuable hints that can direct science development. It's similar to simulation. As a theorist, I always consider simulation as a way to peek at the answer before we develop theories.

As time goes on, I also realize that certain things are only possible when you have a huge amount of data. The vast data enables us to identify and understand things in ways not possible before. As I have mentioned, if you ask the large language models to explain how they came to certain answers, they are able to give explanations. But obviously the current explanations are not good enough to satisfy scientists like us. In terms of explainable AI, there is still a long way to go.

Anyway, AI is changing the way we do science and I think we are at a very early stage of new types of science.

**Wang:** I think one main weakness of the current large language models is that they cannot reason in as a logical way as human beings. That may be why they cannot give explanations to their answers.

**Srolovitz:** That's right. But do you think reasoning like humans can give you better results? I'm not sure about that.

**Car:** I agree that the new data from AI tools can provide insight, but when you get these data, it still requires a human scientist to decide what kind of further analysis needs to be made. That is what AI cannot do.

Indeed, AI is generating a new paradigm for scientific research, but it does not mean the traditional research concepts will be superseded or abandoned. On the contrary, they should be reinforced for the robust validation of the machine learning models. These models allow us to extrapolate the model prediction into contexts that are much wider than those where the training occurred. And it is often difficult to validate the predictions on rigorous mathematical bounds.

For instance, as I mentioned that in simulation, AI provides a bridge between quantum mechanical calculation and molecular simulation. But in large-scale systems with rare events that are essential but not considered in the training data, the AI tools may fail. That is when we need to analyse the problem with the basic tools of physical intuition and physical thinking, and that will facilitate the development of new theoretical models, which eventually will be expressed by new differential equations that can better represent the dynamics of complex systems than do existing models.

**E:** AI often gives an impression of being black magic. I believe this does not have to be the case. At the moment, AI is very much like an experimental or engineering playground. But I think over time, this will change. People will begin to find some guiding principles. In fact, there has already been a lot of progress in this direction, though for some reason, the larger AI community does not seem to be aware of these results. So, I think AI can be also turned into a rather scientific subject.

It is true that AI is better at getting answers than understanding. But again, this does not have to be the case. One example is to use a knowledge graph to understand the relationship between different molecules. I am not sure that this is done, but I would say this is helpful. We created a knowledge graph in economics. It helped to unravel how different economic parameters are related to each other. I am sure something like that should be very helpful and revealing in science.

I had thought of the AI approach as anti-scientific, because it often was about getting answers, but not about getting understanding.

—David Srolovitz

## CAN AI CREATE?

**Car:** In my impression, the AI models can do routine analysis, but find it difficult to do any kind of things that require creativity. But I may be wrong, or I may become wrong in a very short time.

**E:** I think AI can create, but it is too early to get into the details.

**Srolovitz:** The current generative models can create art, and I feel that they are really good at creating art. Is that creativity? I'm not sure. By the way, I always felt that interatomic potentials were a scientific art. It is possible that 15 or 20 years from now, scientific inquiry will not be the way we grew up with.

**Wang:** How I understand the generative art models is that the generated pictures are more or less the combination of the existing art styles in the training data, and they cannot generate totally new art styles. Actually, most of the human artworks are the combination of existing components or ideas of the existing arts, and AI can make good combinations in a creative way.

**Srolovitz:** Even for scientific research, I think most of the inquiry attempts are to combine existing things in new ways.

The generated pictures are more or less the combination of the existing art styles in the training data, and they cannot generate totally new art styles.

—Han Wang

## AN OPEN SYSTEM FOR AI4S

**Zhang:** Who should be the major promoter of AI4S, scientists or technology companies? How could the interested parties cooperate with each other?

**E:** I hope that the scientific community will lead the AI4S revolution. It will take time for the companies to build the necessary scientific background. More importantly, the scientific community has also always been the main force in doing scientific research. In the future, AI will be thoroughly integrated into scientific research. So, if we lose that leadership, we will be in a rather difficult position, just as what has happened in large language models.

**Srolovitz:** Obviously, no research group can afford to create a competitive large language model. As scientists we should not try to write our own version of that. What we ought to do is learning how to harness it, train it and engineer it to do the things we want to do. It's like that no research group tries to create its own hadron collider. It's a tool that scientists use.

**E:** Ultimately this is an issue of resources and outcomes. At the moment, AI4S is still largely a research activity, even though there are clear commercial opportunities. This is a great opportunity for the funding agencies. In this regard, the National Natural Science Foundation of China has done a good job by putting out a major research program to support AI4S.

**Wang:** I feel that the interest of the companies may not be always aligned with that of the scientists. If it cannot produce a profit, the companies will not develop tools for the scientists. That may be the main gap between the scientific community and the companies. This gap may be filled by government investment. But I don't know whether it will be enough.

**Srolovitz:** Nowadays, scientific researchers, even including the researchers of military technologies, are learning to use commodity technologies for the applications they care about. Even though that technology was not developed for them, they will learn how to harness it. So, I think in case the AI technologies are not built for scientists, the challenge for us is to learn how to harness it to do the things we as scientists want to do.

**Zhang:** The development of AI4S tools, such as the DeepMD-kit we have created, is contingent upon the collaborative efforts of numerous community partners. Throughout the development process, the challenges and bottlenecks we encounter tend to shift over time.

Initially, the primary challenge lay in the design of the model and the development of the software. Subsequently, to accommodate the diverse needs of various users, there was a demand for technical personnel who are proficient in software operation and possess a deep understanding of scientific issues. Following this, the infrastructure, including high-performance computing and cloud computing, emerged as the new bottleneck. Currently, having amassed a substantial amount of data, we are presented with the possibility of developing large atomic models; however, this also introduces new challenges in model and software development.

In this process, we are also committed to fostering an open-source community known as DeepModeling and constructing a stable user interface for these tools on a cloud platform called Bohrium. Our aspiration is for this interface to be as intuitive and user-friendly as personal computers or smartphones, enabling users from different backgrounds to freely explore and address their specific problems.

There are always emerging bottlenecks that are jumping from one community to another.

—Linfeng Zhang

**Car:** I'm not sure if we need a full integration of AI4S tools by creating an interface for all the tools, but certainly some further level of integration will occur, and I think it is very beneficial. This will demand more interaction between different sub-communities, including simulators, material designers and experimentalists.

**Srolovitz:** One fact is that scientists are not good at developing the interfaces or the standardized tool sets. What scientists can do better than companies are to provide good questions, which is essential for science itself.

**Car:** We need both the companies and scientists. We need an open system where the exchanges of information can be made easily, where data are accessible to everyone to check, and where new questions can be raised freely. If we can keep that system going, there will be a lot of progress. But unfortunately, there are many difficulties in keeping this system alive.

**Srolovitz:** Right. The openness is crucial. Every time a technology that will change the economy and society emerges, a big disappointment is that different countries would try to corral it to their own advantage. I'm very optimistic about the AI technology and what it can do for science, but such corralling may delay things more than anything else. But it reminds me of the famous line in the movie *Jurassic Park*, ‘Life Finds a Way’. I think we will eventually break out of that situation, and the question is how long it will take.

We need an open system where the exchanges of information can be made easily, where data are accessible to everyone to check, and where new questions can be raised freely.

—Roberto Car

## CHALLENGES AND OPPORTUNITIES

**Zhang:** Thank you for all the discussions. As a conclusion, please give one bottleneck and one suggestion for AI4S.

**Car:** One bottleneck of AI4S in the area of molecular simulation is that it cannot deal with the electron transfer phenomena very well. Electron transfer is essential for all kinds of chemical reactions, but as we cannot yet capture the accurate electronic coordinates, it's still difficult to simulate this phenomenon. To solve this issue, it requires development of not only AI technology, but also new ways of modeling that goes beyond the current scientific paradigm based upon basic physics laws such as the Born-Oppenheimer approximation and the density functional theory.

One suggestion for AI4S is what we have discussed: we need to make every effort in the scientific community to keep the domain open and operate according to the rules and methods of science.

**Srolovitz:** Looking into the future, I'm really interested in seeing more about explainable AI—to be able to understand what's behind the AI predictions. There is a lot of work in this area now and I'm optimistic about it. I also suppose that such progress will probably benefit science more than it will benefit computer science and AI technology.

**Wang:** For me, the next great opportunity would be the large atomic model. The ultimate goal is to build a universal model for the periodic table, but I don't think it will be realized in the predictable future. But the large atomic model, as a pretrained model for atomic simulations, would be feasible in the near future.

The bottleneck of the large atomic model is data sets. The broader the data we have in the training set, the higher the generalizability that would be obtained. But unlike the large language models that can obtain language materials from everywhere, the training data required by large atomic models is very limited and very expensive, such as the fine crystal structures and the DFT calculation results.

**E:** The biggest bottleneck is the lack of good data. One suggestion is to use new AI-based models to generate high-quality data. To make that happen, we still have to work on our simulation capability. For example, although AI-based algorithms have greatly improved our ability to perform molecular dynamics simulations with *ab initio* accuracy, there is still a substantial gap from being able to use these new tools to simulate real systems of interest, due to the fact that the time scale we can simulate is still limited and the modeling of defects is still quite expensive.

## Notes

Weijie Zhao is an NSR science and news editor based in Beijing.

